# Short-Term Environmental Enrichment is a Stronger Modulator of Brain Glial Cells and Cervical Lymph Node T Cell Subtypes than Exercise or Combined Exercise and Enrichment

**DOI:** 10.1007/s10571-020-00862-x

**Published:** 2020-05-25

**Authors:** Gaurav Singhal, Julie Morgan, Frances Corrigan, Catherine Toben, Magdalene C. Jawahar, Emily J. Jaehne, Jim Manavis, Anthony J. Hannan, Bernhard T. Baune

**Affiliations:** 1 Psychiatric Neuroscience Lab, Discipline of Psychiatry, The University of Adelaide, Adelaide, Australia; 2 Division of Health Sciences, The University of South Australia, Adelaide, Australia; 3 School of Psychology and Public Health, La Trobe University, Bundoora, Melbourne, Australia; 4 Centre for Neurological Diseases, School of Medicine, Faculty of Health, The University of Adelaide, Adelaide, Australia; 5 Florey Institute of Neuroscience and Mental Health, The University of Melbourne, Melbourne, Australia; 6 Department of Psychiatry, Melbourne Medical School, The University of Melbourne, Melbourne, Australia; 7 Department of Psychiatry, The University of Münster, Münster, Germany

**Keywords:** Environmental enrichment, Physical exercise, Aging, Microglia, Astrocytes, T cells

## Abstract

Physical exercise (PE) and environmental enrichment (EE) can modulate immunity. However, the differential effects of short-term PE, EE, and PE + EE on neuroimmune mechanisms during normal aging has not been elucidated. Hence, a cohort of 3-, 8-, and 13-month-old immunologically unchallenged C57BL/6 wild-type mice were randomly assigned to either Control, PE, EE, or PE + EE groups and provided with either no treatment, a running wheel, a variety of plastic and wooden objects alone or in combination with a running wheel for seven weeks, respectively. Immunohistochemistry and 8-color flow cytometry were used to determine the numbers of dentate gyrus glial cells, and the proportions of CD4^+^ and CD8^+^ T cell numbers and their subsets from cervical lymph nodes, respectively. An increase in the number of IBA1^+^ microglia in the dentate gyrus at 5 and 10 months was observed after EE, while PE and PE + EE increased it only at 10 months. No change in astroglia number in comparison to controls were observed in any of the treatment groups. Also, all treatments induced significant differences in the proportion of specific T cell subsets, i.e., CD4^+^ and CD8^+^ T naïve (T_N_), central memory (T_CM_), and effector memory (T_EM_) cells. Our results suggest that in the short-term, EE is a stronger modulator of microglial and peripheral T cell subset numbers than PE and PE + EE, and the combination of short-term PE and EE has no additive effects.

**Electronic supplementary material:**

The online version of this article (10.1007/s10571-020-00862-x) contains supplementary material, which is available to authorized users.

## Introduction

The detrimental effects of aging on the neuroimmune system are well documented (Montecino-Rodriguez et al. 2013; Thoman and Weigle 1982). During normal aging, the immune system becomes increasingly sensitized to both intrinsic and extrinsic pathogenic factors and shows a constant functional decline, impacting health and survival. Studies have shown that microglia and astrocytes, which are essential for the innate neuroimmune and tissue repair processes and the maintenance of neurobiological homeostasis (Hanisch 2002; Rothwell et al. 1996), play a role in the onset of inflammatory and degenerative changes in the brain during aging (Conde and Streit 2006; Schipper 1996). The glial cells of the brain become increasingly dysfunctional, lose neuroprotective properties, and secrete significantly higher levels of proinflammatory cytokines (e.g., TNF-α, IL-1β) and neurotoxic substances (e.g., reactive oxygen species, nitric oxide) during aging, which contributes to chronic neuroinflammation and subsequent neurobiological damage, cognitive dysfunction, memory deficit, and mood disorders (Mrak 2012; Patel 2013). The increased activation of hippocampal IBA^+^ microglia and GFAP^+^ astrocyte cells with the increased expression of IL‐1β during aging has been reported to be responsible for neuroinflammation-associated impaired neurogenesis and loss of brain function (Kuzumaki et al. 2010).

Circulating T cells, which are important for cell-mediated immunity, as well as for the healthy functioning of the brain and the maintenance of neuroimmune bidirectional communication and homeostasis (Ron-Harel et al. 2011), undergo a major transformation during aging. Evidence suggests that there is an aging-induced reduction in the thymic output that results in a low number of naïve T cells (Montecino-Rodriguez et al. 2013). As a result, while the proliferation of T cells continues during aging, there is a reduction in the ability to establish immunological memory in response to new antigens. Furthermore, there is a gradual decline in the number of early T cell activation markers and increase in the number of memory T cells_,_ suggestive of the adverse effects of aging on cell-mediated immunity (Moro-García et al. 2013). The significant reduction in the antigen presentation capacity of the helper CD4^+^ T cells and antigen elimination function of the cytotoxic CD8^+^ T cells has also been reported during normal aging (Gruver et al. 2007; Linton and Dorshkind 2004). Collectively, the alteration in peripheral T cell subsets during aging can predispose the brain for neuroinflammatory and neurodegenerative diseases.

The abovementioned dysregulated innate and adaptive immunity makes the aging brain prone to increased occurrence of lymphoproliferative disorders, the formation of neurotoxic amyloid-beta and tau proteins, and expression of proinflammatory cytokines and acute-phase proteins. The resultant exacerbated neuroinflammation, in turn, may act as an etiology for neurodegenerative disorders during old age, including Alzheimer’s disease (AD) and dementia (Eikelenboom et al. 2006). For this reason, several pharmacological and non-pharmacological treatments have been explored in recent times to influence the aging-associated neuroimmune changes in the brain. Evidence suggests that non-pharmacological treatments, such as physical exercise (PE) and species-specific enrichment of the external environment (environmental enrichment, EE), exert a considerable influence on behavior and neuroimmune mechanisms (Baumans 2005; Singhal et al. 2014), and therefore could be utilized to modulate the neuroimmune functions associated with aging-related brain disorders. Indeed, in animal studies, these treatments (i.e., PE and EE) can be studied separately and in combination.

There are substantial evidence suggesting that hippocampus, particularly the dentate gyrus region, play a vital role in the regulation of behavior and memory in response to external stimulus (Engin and Treit 2007; Sampedro-Piquero et al. 2013; Schacter et al. 1996; Scoville and Milner 1957). Both PE and EE have been shown to enhance hippocampal neurogenesis, improve cognition, memory, and motor coordination, and alleviate mood disorders in preclinical rodent models of psychiatric disorders (Ahmadiasl et al. 2003; Binder et al. 2004; Faherty et al. 2005; Falkenberg et al. 1992; Hannan 2014; Jankowsky et al. 2005; Kempermann et al. 2002; Nichol et al. 2009; Van der Borght et al. 2007; Van Praag et al. 2005). Research has also shown that the beneficial effects of PE and EE on brain functions are the result of altered expression of peripheral T cells (Marashi et al. 2003, 2004) and brain glial cells (Chabry et al. 2015; Ehninger and Kempermann 2003; Williamson et al. 2012). Together, this suggests that PE and EE modulate brain functions by also altering the expression of T cells in the periphery and glial cells in the dentate gyrus region of the hippocampus in the brain.

The paradigms of PE and EE function either at the physical or cognitive level. In PE, rodents are provided with voluntary or involuntary access to run on a wheel or treadmill, respectively, as compared with EE where mice voluntarily engage with cognitive stimuli including a range of objects of different sizes, shapes, and composition (e.g., plastic tubes, toys, ropes, ladders, tunnels, house, ramps, and platforms). It is, however, important to note that running wheels have also been used as an EE tool in a large number of studies (Berardi et al. 2007; Jankowsky et al. 2005; Kempermann et al. 2002; Leggio et al. 2005; Moncek et al. 2004; Morley‐Fletcher et al. 2003; Segovia et al. 2006; Williamson et al. 2012). Since immune alterations in the brain during aging can affect behavior, mood, and cognition (Dantzer et al. 2008; Maier and Watkins 1998), this, therefore, also suggests that short-term PE, EE, and their combination could be useful as immunotherapy for age-related brain disorders.

The reported neuroimmune outcomes after short-term PE and EE, however, have been inconsistent across past studies. For instance, EE of 8 to 16 weeks has been shown to reduce the levels of cytokines IFN-γ, TNF-α, IL-2, and IL-10 and the percentage of CD8^+^ cells in the supernatants of activated spleen cells, and improve macrophage chemotactic activity and phagocytosis, and basal lymphocyte proliferation and chemotactic activity in mice (Arranz et al. 2010; Marashi et al. 2003, 2004). Short-term PE alone has been shown to improve neuroimmune functions in rodents by enhancing NK cell activity and T suppressor cell activity (Benaroya‐Milshtein et al. 2004; Kaufman et al. 1994). Furthermore, the combination of short-term PE and EE (PE + EE) has been shown to increase astrocyte and microglia antigen expression in the dentate gyrus, and decrease the expression of proinflammatory cytokines TNF-α and IL-1β in the hippocampus of Sprague Dawley rats (Williamson et al. 2012). These disparate immune outcomes, therefore, create uncertainty regarding the utility of short-term PE and EE as external non-pharmacological treatments in psychiatric studies. Also, the neuroimmune outcomes may vary across the life span. For example, a preclinical study in rodents has shown that while short-term EE increases the astrocyte number and size, as well as GFAP levels, in the hippocampus and corpus callosum of young male Wistar rats, it decreases the same in old rats (Soffié et al. 1999).

The currently available literature, therefore, lacks evidence that can establish the differential neuroimmune effects of short-term PE, EE, and their combination across the lifespan. In our recently published study (Singhal et al. 2019), we found that the short-term EE of 4 weeks in the absence of PE improved locomotion, and reversed age-related anxiety and cognitive deficit, but showed no significant effects on depressive-like behavior. Short-term PE and PE + EE, however, were found to be ineffective. We, therefore, wanted to investigate whether the observed changes in behavior are related to underlying immune changes. Hence, all mice were sacrificed the day after behavioral testing ended for the analyses of change in numbers of astrocytes and microglia in the dentate gyrus and T cell subset proportions from cervical lymph nodes. C57BL/6 mice were selected for the study since they have been characterized well in terms of humoral and cellular neuroimmune responses to environmental factors (Song and Hwang 2017). The dentate gyrus region of the hippocampus was selected for molecular analysis due to its known role in the regulation of behavior and memory as mentioned previously. Since the migration of leucocytes from brain to cervical lymph nodes is established (Goldmann et al. 2006) and it has been suggested that T cells from the cervical lymph nodes are good representative of the status of cellular immunity in the brain (Engelhardt et al. 2016), we selected cervical lymph nodes for the analysis of T cell-mediated immunity in the brain of mice for our research.

## Methods

### Animals

Wild-type (C57BL/6) mice (*n* = 158; 80 males and 78 females), parental substrain Nhsd (derived from a colony from the National Institutes of Health, Bethesda, Maryland, USA), were bred in-house in the laboratory animal services (LAS) facility at the University of Adelaide and housed in same-sex groups of 4–5 in individually ventilated cages (IVCs) under controlled conditions of temperature (21 ± 1 °C), humidity (55%), and a 12–12-h dark–light cycle. During experiments, the C57BL/6Nhsd line was inbred for 9–13 generations.

### Experimental Design

**Fig. 1:**
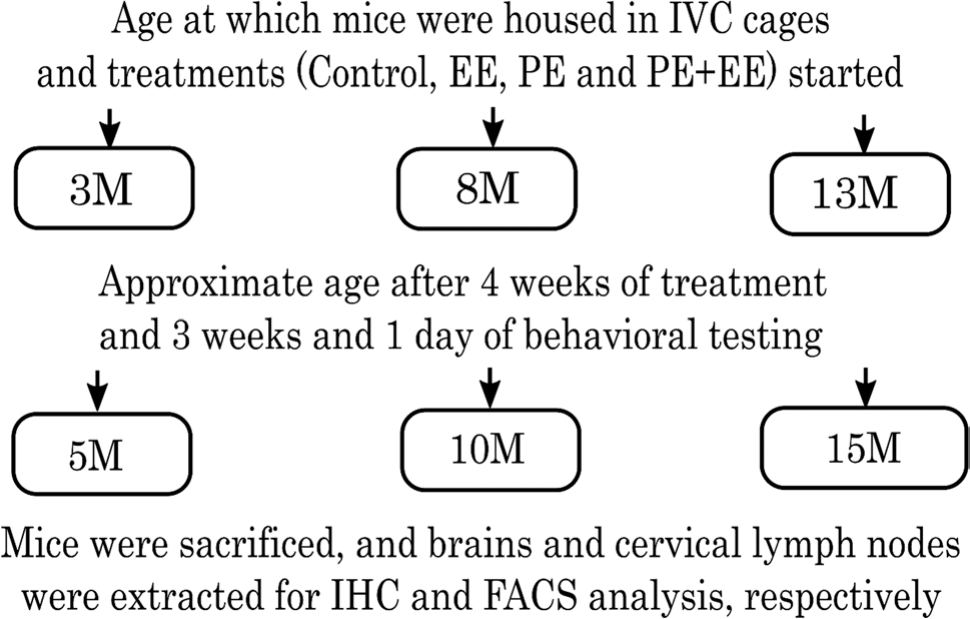
Schematic representation of the experimental design. *M* months, *W* weeks, *IHC* Immunohistochemistry, *FACS* Fluorescence-activated cell sorting

Early age (3 m), middle age (8 m), or late-middle age (13 m) mice that showed no signs of injury and sickness, hence not challenged immunologically, were randomly allocated into four groups, i.e., Control, Physical Exercise (PE), Environmental Enrichment (EE), and PE + EE, as shown in Fig. 1 and Supplementary Fig. 1. Mice were then randomly paired (males and females paired separately) and transferred to open top cages on the morning of Monday, week 1 (2 mice per cage unless fighting necessitated separation). Each group had 11–19 (50% male and 50% female) mice per age group. Control mice received no treatment and were kept in cages with the following dimensions: 48.5 cm × 15.5 cm × 12 cm. PE, EE, and PE + EE mice were kept in plexiglass cages with dimensions: 37 cm × 20.5 cm × 13.5 cm, as these cages provided more breadth and depth to provide extra space for the objects associated with EE and running wheels.

The groups assigned as PE and PE + EE were provided with a running wheel for 4 weeks before behavioral testing, i.e., starting at 3, 8, and 13 months of age and ending at 4, 9, and 14 months of age, respectively. Similarly, the groups assigned as EE and PE + EE were provided with a variety of non-toxic novel objects (houses, colored balls, toys, hanging toys, ladders, and tunnels) and extra bedding as per previously published protocols (Jankowsky et al. 2005; Leggio et al. 2005; Spires et al. 2004) for 4 weeks. The running wheels and EE objects remained in the cages throughout the three weeks of the behavioral testing period that followed four weeks of treatments (Fig. 1). The ages of the mice at the end of behavioral testing (3, 8, and 13 months + 7 weeks, respectively) are referred as 5, 10, and 15 months throughout the paper for the sake of simplicity.

Mice were inspected daily but handled only once a week while transferring them to clean cage every Friday morning to minimize handling stress, starting Friday of week 1. At the same time, mice were weighed on a digital weighing scale and wheel revolutions were counted using an automated counter on the wheels, the analyses of which has been published (Singhal et al. 2019). Also, the wheels were cleaned with F10SC veterinary disinfectant and the EE objects were changed to maintain novelty for mice. Mice were monitored for dominancy throughout the experiments, and those found to be highly aggressive were segregated to prevent dominance effects on neuroimmune mechanisms. Only the EE mice received nesting material (paper shreds) during the experiments. Mice underwent 3 weeks and 1 day of behavioral testing post four weeks of treatment (Fig. 1).

## Molecular Analyses

After seven weeks exposure to treatments during which all mice also underwent identical behavioral testing in the last three weeks, mice were euthanized with a lethal dose of pentobarbital (60 mg/kg IP), and blood was collected through cardiac puncture (Parasuraman et al. 2010). Mice used for immunohistochemistry were perfused with 10% neutral buffered formalin via transcardiac injection, with the brains rapidly removed and preserved in 10% formalin. Mice utilized for FACS have had their draining cervical lymph nodes of the brain removed and collected in Roswell Park Memorial Institute (RPMI+) medium. It is important to note that the behavioral testing ended with the forced swim test a day before tissue collection. For full behavioral testing protocol and data analyses, see our recently published paper (Singhal et al. 2019). While both male and female mice were used to represent findings from a population comprising of both sexes in approximately equal numbers, it is important to note that we could not perform sex analysis due to low n for both males and females when taken separately.

### Analysis of the Number of Immunopositive Glial Cell Markers (IBA1 and GFAP) in the Dentate Gyrus

Following collection in 10% formalin, brains were cut into five 3 mm coronal slices and following overnight treatment with increasing concentrations and exposure of ethanol, xylene, and paraffin baths; the sliced brain samples were embedded in paraffin wax. The hippocampus was then serially sectioned, with six sections 150 μm apart. Each section was 5 μm thick.

For immunohistochemistry, on day 1, sections were dewaxed and dehydrated in xylene and ethanol, and endogenous peroxidase activity was blocked by incubation with 0.5% hydrogen peroxide in methanol for 30 min. Antigen retrieval was performed by heating at close to boiling point for 10 min in citrate buffer, and slides were then allowed to cool below 40 °C before further processing. The appropriate primary antibody (IBA1 for microglia, 1: 10,000; GFAP for astrocytes 1: 40,000; Abcam) was applied to the slides which were then left to incubate overnight for allowing primary antibodies to bind to the target antigen. On day 2, the IgG biotinylated antibody of rabbit (same as primary antibodies) was added and allowed to react with primary antibodies for 30 min. The formed immune complex was then further amplified by incubating slides with a biotin-binding protein, streptavidin-peroxidase conjugate, for 60 min. The immune complex was then visualized with precipitation of DAB in the presence of hydrogen peroxide. Slides were washed to remove excess DAB and lightly counterstained with hematoxylin, dehydrated, and mounted with DePeX.

All slides were digitally scanned using the Nanozoomer, (Hamamatsu City, Japan) and then viewed with the associated software NDP view (version 1.2.2.5). Immunopositive cells in the dentate gyrus region of the hippocampus were counted manually for statistical analysis. Freehand boxes were drawn to cover the entire dentate gyrus regions of the six stained sections followed by counting of the cells within the boxes. For each section, the total number of cells was then divided with the area of the box (in mm^2^) to get the number of cells/mm^2^. The average of six sections represented the value for one mouse and was utilized during statistical analysis.

### Peripheral T Cell Immunophenotyping Using Fluorescence-Activated Cell Sorting (FACS)

For the analysis of T cell-mediated immunity, we extracted the cervical lymph nodes and total number of T cells, CD4^+^ and CD8^+^ T cell subpopulations (Naïve or T_N_, Central memory or T_CM_ and Effector memory or T_EM_), and early-activated T cell phenotype CD25 were characterized using FACS.

Cervical lymph nodes were retrieved one day after behavioral testing ended and collected in RPMI + medium. Lymph nodes were passed through a 0.1 µ sieve (BD) using RPMI + and the end of a 1 mL syringe and centrifuged to separate cells from tissue debris. Retrieved lymph node cells were counted on a hemocytometer and re-suspended in PBS to a final concentration of 2 × 10^6^ cells/ml. 250 µL of the cell solution was then washed once with FACS buffer (PBS with 1% heat-inactivated bovine serum albumin) and blocked with 10 µL 0.5 mg/mL Fc block. Eight color staining panel was used to immunophenotype CD4^+^ and CD8^+^ T cells. Unstained cells were used to exclude autofluorescent cells, while single stained and fluorescence minus one (FMO) stained cells were used to control for spectral overlap or distinguishing between negative and positive cells, respectively (non-specific binding). Cells were incubated for 30 min at room temperature with the respective mAbs (as shown in Table 1) after which they were washed twice before resuspension in 300µL FACS buffer. Cells were analyzed using the Gallios flow cytometer, and 100,000 events were acquired. The data obtained were analyzed using FCS Express software (version 4). Forward side scatter gating on acquired data distinguished singlet from doublet cell populations from which CD45^+^ cells were gated. Percentages of CD3^+^ CD4^+^ or CD3^+^ CD8^+^ gated cells were used to calculate total cell numbers in combination with cell counts. Further gating on CD44^+^ and CD62L^+^ cell populations in the FCS Express software enabled identification and estimation of T cell subsets (T_N_, T_CM_, and T_EM_).

**Table 1:** Monoclonal antibodies used for T cell immunophenotyping

mAb	Clone	Fluorochromes	BD biosciences catalog no	Conc. (mg/mL)	Antigen distribution/function
CD3	145-2C11	FITC	553061	1.0 × 10^3^	T cell identification marker
CD45	30-F11	V500	561487	2.0 × 10^3^	Nucleated hematopoietic cell lineage marker; common leukocyte antigen
CD4	GK1.5	APC-H7	560181	2.0 × 10^4^	T helper cell co-receptor for MHC II-restricted antigen induced T cell activation
CD8	53–6.7	PerCP-Cy5.5	551162	2.0 × 10^4^	Cytotoxic T cell Co-receptor for MHC I restricted antigen induced T cell activation
CD25	3C7	PE	561065	2.0 × 10^3^	Early T cell activation marker
CD44	IM7	PerCy7	560569	5.0 × 10^4^	Activation marker for effector or memory T cell; attachment and rolling
CD62L	MEL-14	V450	560507	5.0 × 10^5^	T cell homing receptor; transmigration
CD69	H1.2F3	APC	560689	2.0 × 10^3^	Early T cell activation marker

*mAb* monoclonal antibody, *Conc*. concentration, *MHC* major histocompatibility complex

### Statistical Analysis

Statistical analyses were conducted using GraphPad Prism version 7.02 (GraphPad Software Inc.). All data outliers were removed using the ROUT method, and normality of data distribution was determined by visual inspection of histograms. Comparisons between the treatments (PE, EE, PE + EE) and controls were performed using two-way ANOVA. The multiple comparisons post hoc Holm–Sidak’s test was used to confirm significant interactions between groups. Results are presented as mean ± SEM. Differences are considered statistically significant when *p* < 0.05.

## Results

### Alteration in Brain Microglia and Astrocyte Number in C57BL/6 Mice Exposed to PE, EE, or PE + EE at 5, 10, and 15 months of Age

**Fig. 2:**
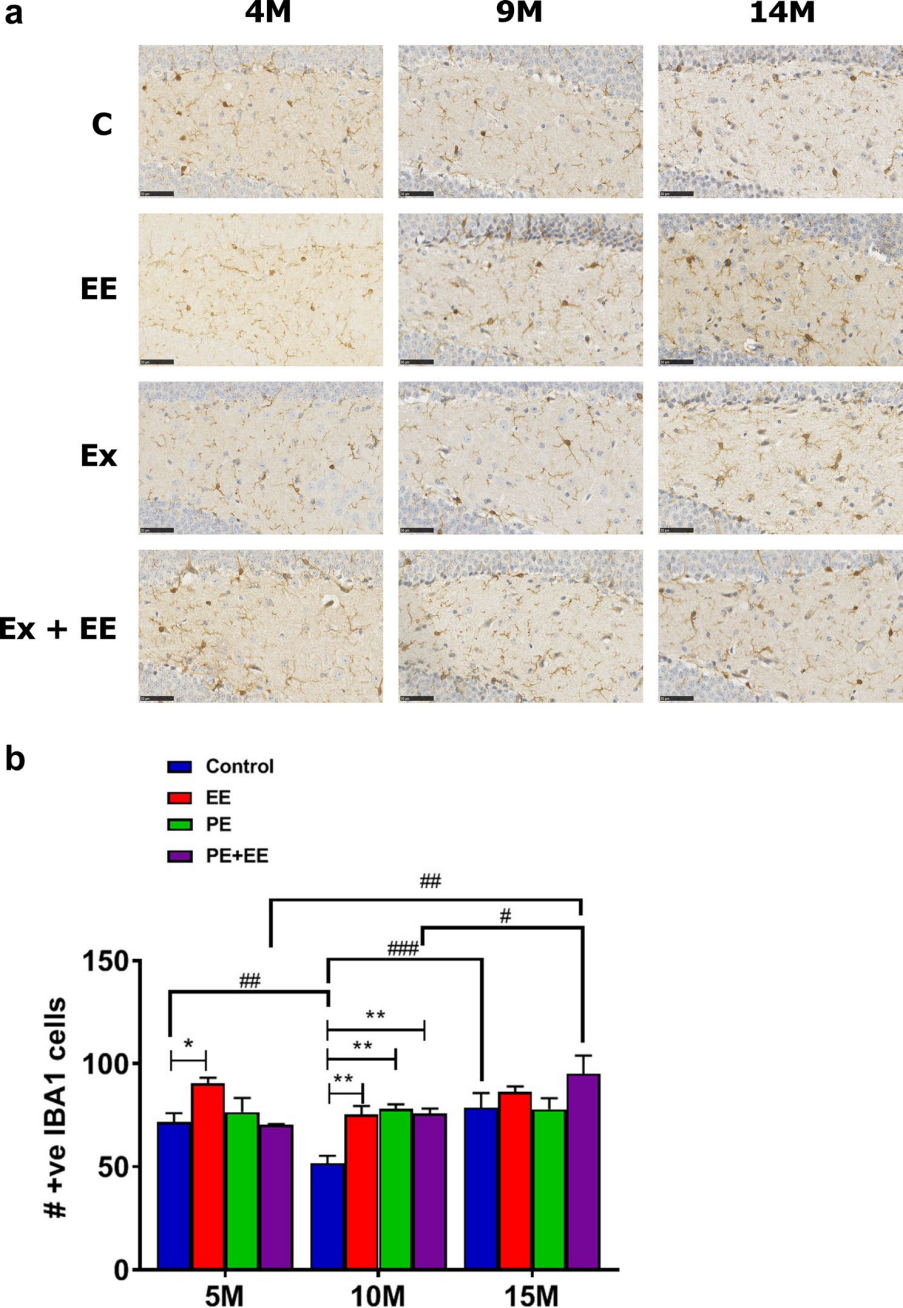
IBA1^+^ microglia in DG. **a** Representative immunohistochemical images of the number of IBA1^+^ microglia in the dentate gyrus of 5-, 10-, and 15-month cohorts of animals exposed to control, PE, EE, and PE + EE conditions (black scale represents 50 µm length), and **b** number of IBA1^+^ microglia cells where data are represented as mean ± SEM, *n* = 4–8 per group. * represents a significant difference between a treatment and age-matched control or between two treatments at one age point. ^#^Represents a significant difference between the matched treatments over two age points. *^,#^* p* < 0.05, **^,##^* p* < 0.01, ^###^* p* < 0.001

Immunopositive (IBA1^+^) microglia were counted in the dentate gyrus of the hippocampus and analyzed statistically using two-way ANOVA with post hoc Holm–Sidak’s multiple comparison test (Fig. 2). A significant interaction effect (*F*_(6, 56)_ = 2.936; *p* < 0.05) with significant main effects of treatment (*F*_(3, 56)_ = 6.749; *p* < 0.001) and age (*F*_(2, 56)_ = 8.947; *p* < 0.001) were noted. EE-treated mice showed a significantly higher number of IBA1^+^ microglia than control mice at both 5 (90.3 ± 2.9 vs. 71.6 ± 4.4; *p* < 0.05) and 10 months (75.4 ± 4.2 vs. 51.7 ± 3.6; *p* < 0.01) of age. Also, PE- and PE + EE-treated mice showed a significantly higher number of IBA1^+^ microglia than control mice at 10 months (78.0 ± 2.3 and 75.8 ± 4.2 vs. 51.7 ± 3.6; *p* < 0.01). Post hoc analysis for the effect of age revealed that 10-month controls had a significantly lower number of IBA1^+^ microglia in the dentate gyrus than both 5-month-old (51.7 ± 3.6 vs. 71.6 ± 4.4; *p* < 0.01) and 15-month-old (51.7 ± 3.6 vs. 78.7 ± 7.1; *p* < 0.001) control mice. Likewise, 15-month PE + EE-treated mice had a significantly higher number of IBA1^+^ microglia in the dentate gyrus than both 5-month (95.1 ± 8.8 vs. 70.1 ± 0.6; *p* < 0.01) and 10-month (95.1 ± 8.8 vs. 75.8 ± 2.4; *p* < 0.05) PE + EE mice.

**Fig. 3:**
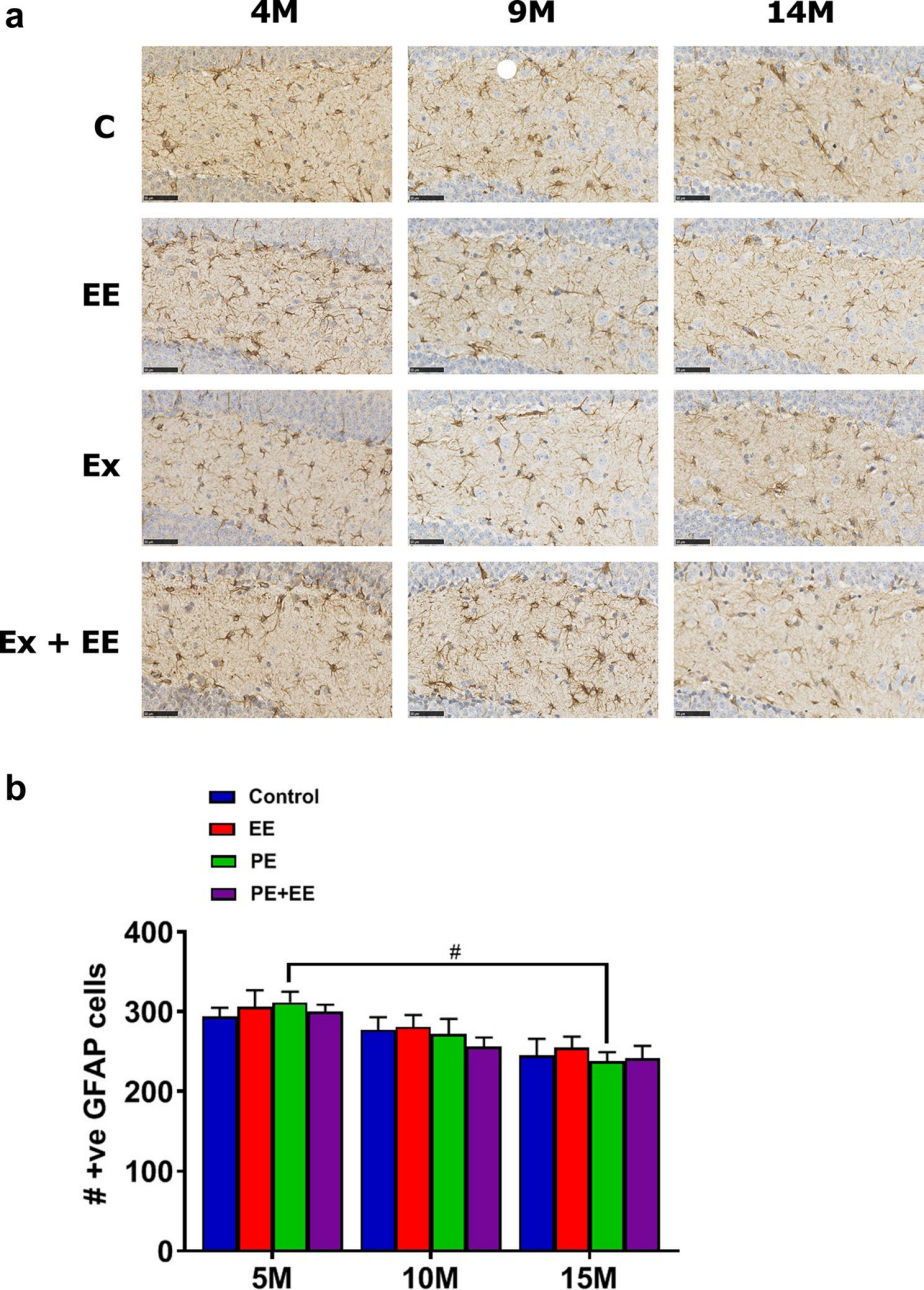
GFAP^+^ astrocytes in DG. **a** Representative immunohistochemical images of the number of GFAP^+^ astrocytes in the dentate gyrus of 5-, 10-, and 15-month cohorts of animals exposed to control, PE, EE, and PE + EE conditions (black scale represents 50 µm length), and **b** number of GFAP^+^ astrocyte cells where data are represented as mean ± SEM, *n* = 4–8 per group. ^#^Represents a significant difference between the matched treatments over two age points. ^#^* p* < 0.05

Immunopositive (GFAP^+^) astrocytes were counted in the dentate gyrus of the hippocampus and analyzed statistically using two-way ANOVA with post hoc Holm–Sidak’s multiple comparison test (Fig. 3). Non-significant interaction effect and main effect of treatment were noted; however, the main effect of age was found to be significant (*F*_(2, 56)_ = 12.73; *p* < 0.0001). This was because, at 5 months of age, PE-treated mice had a significantly higher number of GFAP^+^ astrocytes in the dentate gyrus than their cohort at 15 months of age (311.8 ± 13.4 vs. 237.9 ± 11.7; *p* < 0.05).

### T Cell Immunophenotyping of Cervical Lymph Node Cells Exposed to PE, EE or PE + EE at 5, 10, and 15 Months of Age

**Fig. 4:**
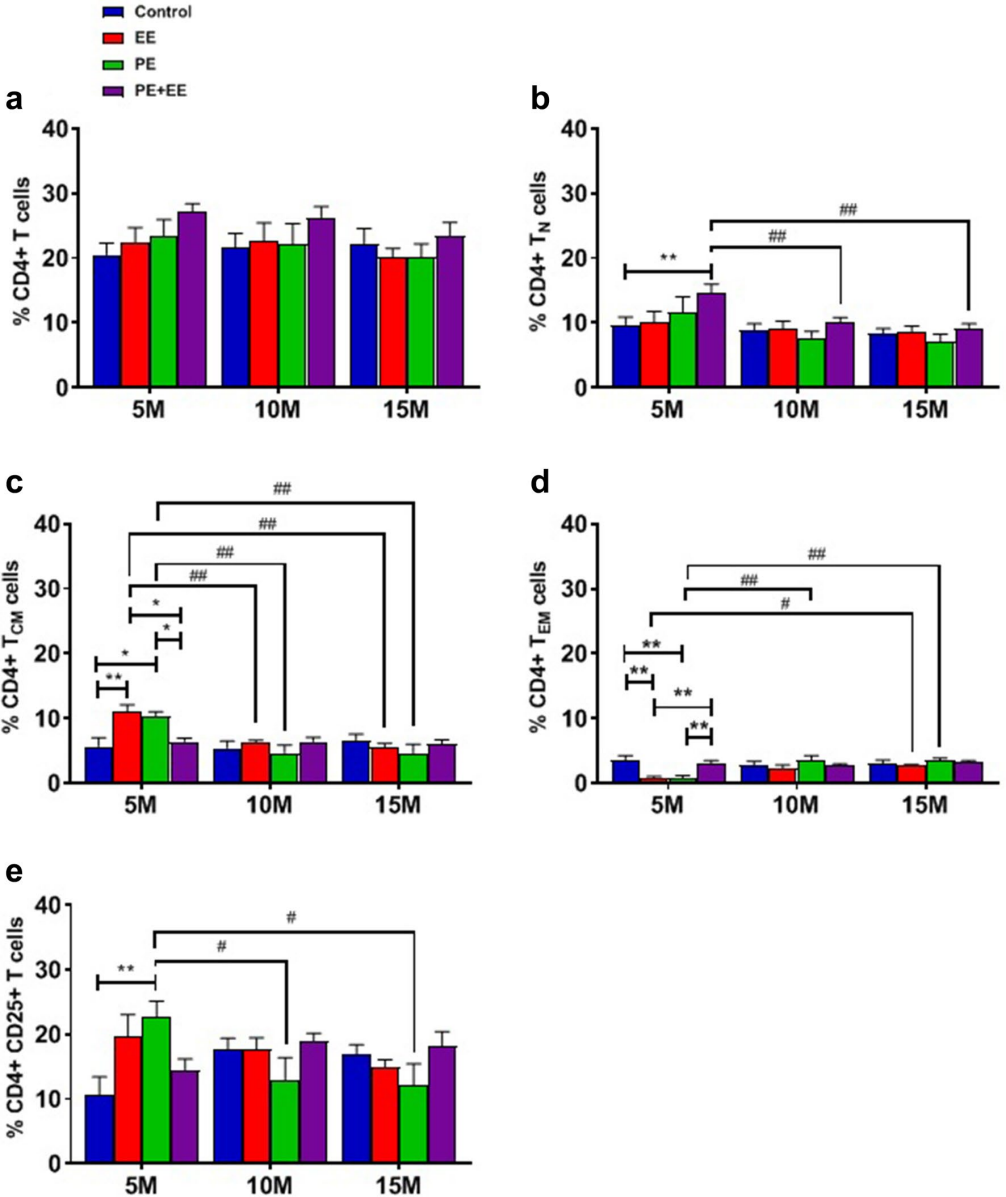
Proportions of the gated **a** CD4^+^, **b** CD4^+^ naïve, **c** CD4^+^ central memory, **d** CD4^+^ effector memory, and **e** CD4^+^ CD25^+^ T cells from cervical lymph nodes of 5-, 10- and 15-month-old C57BL/6 mice with or without treatment. Two-way ANOVA in GraphPad Prism. All data represented mean ± SEM, *n* = 4–8 per group. * represents a significant difference between a treatment and age-matched control or between two treatments at one age point. ^#^ represents a significant difference between the matched treatments over two age points. * and ^#^* p* < 0.05, ** and ^##^* p* < 0.01. *M* month, *EE* environmental enrichment, *PE* physical exercise, *N* naïve, *CM* central memory, *EM* effector memory

**Fig. 5:**
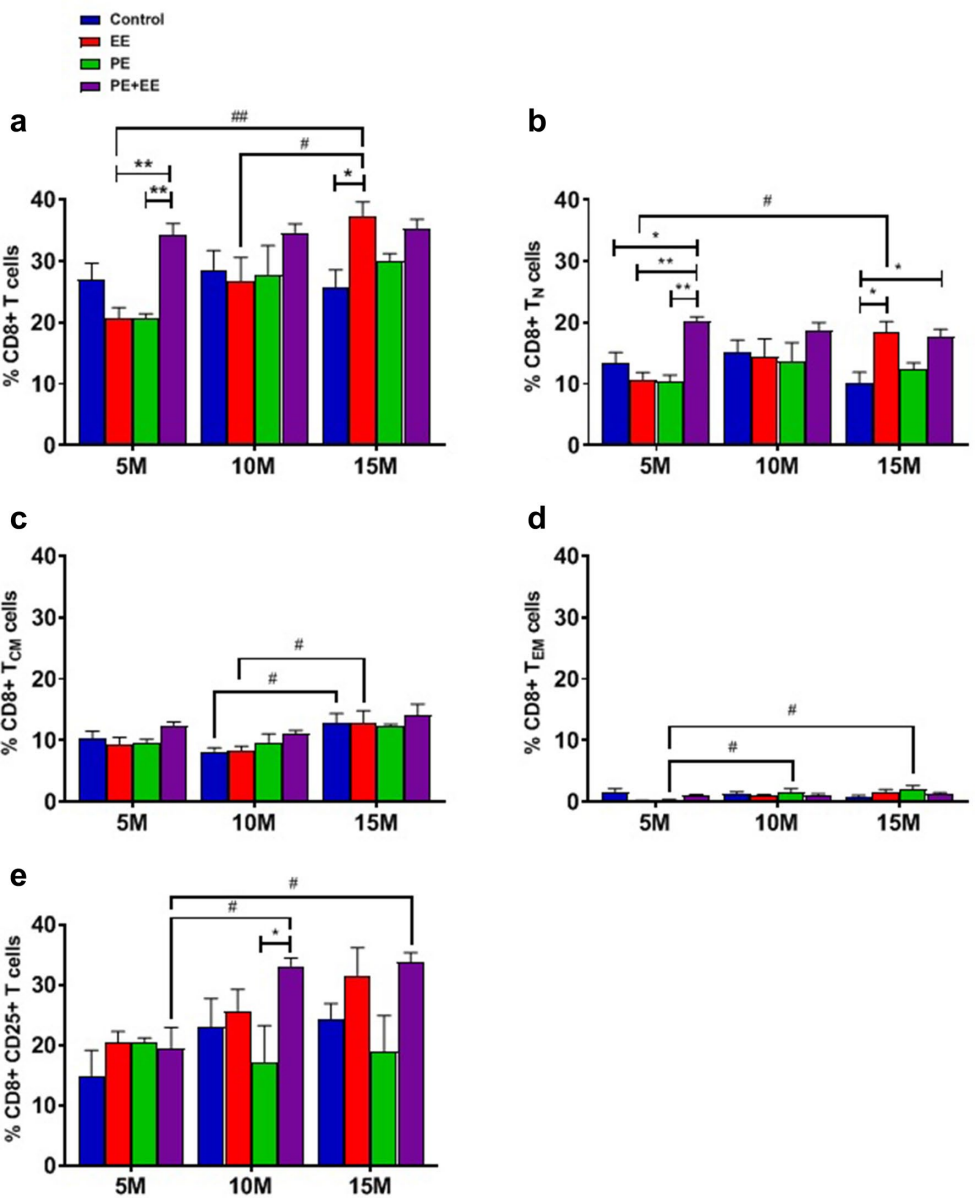
Proportions of gated **a** CD8^+^, **b** CD8^+^ naïve, **c** CD8^+^ central memory, **d** CD8^+^ effector memory, and **e** CD8^+^ CD25^+^ T cells from cervical lymph nodes of 5-, 10- and 15-month-old C57BL/6 mice provided with either no treatment (controls), PE, EE, or PE + EE. Two-way ANOVA in GraphPad Prism. All data represented mean ± SEM, *n* = 4–8 per group. * represents a significant difference between a treatment and age-matched control or between two treatments at one age point. ^#^ represents a significant difference between the matched treatments over two age points. * and ^#^* p* < 0.05, ** and ^##^* p* < 0.01. *M* month, *EE* environmental enrichment, *PE* physical exercise, *N* naïve, *CM* central memory, *EM* effector memory

CD4^+^ (helper T) and CD8^+^ (cytotoxic T) total cell numbers indicate the treatment-specific immune response. Representative images obtained during the FACS data analysis using FCS Express software are shown in Supplementary Figures II and III. The raw FACS data are provided in the attached supplementary excel sheet. Statistical data and group comparisons for T cell subsets (T_N_, T_CM_, and T_EM_) are displayed in Table 2, which are supported by Figs. 4a–e and 5a–e.

**Table 2:** Statistical data and group comparisons for T cell subsets percentage in the cervical lymph nodes of C57BL/6 mice after short-term PE, EE, and PE + EE treatments

T cell phenotype	Interaction effect	Main effect of age	Main effect of treatment	Vs. Control (%)	Between age groups (%)	Between treatment groups (%)
CD4^+^ cells (Fig. 4a)	NS	NS	NS	NS	NS	NS
CD4^+^ T_N_ cells (Fig. 4b)	NS	S (*F*_(2, 60)_ = 7.445; *p* < 0.01)	S (*F*_(3, 60)_ = 3.024; *p* < 0.05)	5 m PE + EE > 5 m C (14.6 ± 1.4 vs. 9.7 ± 1.2; *p* < 0.01)	5 m PE + EE > 10 m PE + EE (14.6 ± 1.4 vs. 10.1 ± 0.7; *p* < 0.01) 5 m PE + EE > 15 m PE + EE (14.6 ± 1.4 vs. 9.2 ± 0.7; *p* < 0.01)	NS
CD4^+^ T_CM_ cells (Fig. 4c)	S (*F*_(6, 62)_ = 3.497; *p* < 0.01)	S (*F*_(2, 62)_ = 9.335; *p* < 0.001)	NS	5 m EE > 5 m C (11.1 ± 1.0 vs. 5.7 ± 1.4; *p* < 0.01) 5 m PE > 5 m C (10.3 ± 0.7 vs. 5.7 ± 1.4; *p* < 0.05)	5 m EE > 10 m EE (11.1 ± 1.0 vs. 6.2 ± 0.4; *p* < 0.01) 5 m EE > 15 m EE (11.1 ± 1.0 vs. 5.6 ± 0.5; *p* < 0.01) 5mPE > 10 m PE (10.3 ± 0.7 vs. 4.7 ± 1.2; *p* < 0.01) 5 m PE > 15 m PE (10.3 ± 0.7 vs. 4.6 ± 1.4; *p* < 0.01)	5 m EE > 5 m PE + EE (11.1 ± 1.0 vs. 6.4 ± 0.5; *p* < 0.05) 5 m PE > 5 m PE + EE (10.3 ± 0.7 vs. 6.4 ± 0.5; *p* < 0.05)
CD4^+^ T_EM_ cells (Fig. 4d)	S (*F*_(6, 58)_ = 3.399; *p* < 0.01)	S (*F*_(2, 58)_ = 4.931; *p* < 0.05)	S (*F*_(3, 58)_ = 3.324; *p* < 0.05)	5 m EE < 5 m C (0.7 ± 0.3 vs. 3.5 ± 0.7; *p* < 0.01) 5 m PE < 5 m C (0.8 ± 0.4 vs. 3.5 ± 0.7; *p* < 0.01)	5 m EE < 15 m EE (0.7 ± 0.3 vs. 2.7 ± 0.2; *p* < 0.05) 5 m PE < 10 m PE (0.8 ± 0.4 vs. 3.6 ± 0.6; *p* < 0.01) 5 m PE < 15 m PE (0.8 ± 0.4 vs. 3.4 ± 0.3; *p* < 0.01	5 m EE < 5 m PE + EE (0.7 ± 0.3 vs. 3.1 ± 0.4; *p* < 0.01) 5 m PE < 5 m PE + EE (0.8 ± 0.4 vs. 3.1 ± 0.4; *p* < 0.01)
CD4^+^ CD25^+^ cells (Fig. 4e)	S (*F*_(6, 61)_ = 3.235; *p* < 0.01)	NS	NS	5 m PE > 5 m C (22.7 ± 2.4 vs. 10.7 ± 2.7; *p* < 0.01)	5 m PE > 10 m PE (22.7 ± 2.4 vs. 13.0 ± 3.4; *p* < 0.05) 5 m PE > 15 m PE (22.7 ± 2.4 vs. 12.1 ± 3.4; *p* < 0.05)	NS
CD8^+^ cells (Fig. 5a)	S (*F*_(6, 61)_ = 2.286; *p* < 0.05)	S (*F*_(2, 61)_ = 5.321; *p* < 0.01)	S (*F*_(3, 61)_ = 6.713; *p* < 0.001)	15 m EE > 15 m C (37.4 ± 2.3 vs. 25.7 ± 2.9; *p* < 0.05)	15 m EE > 5 m EE (37.4 ± 2.3 vs. 20.6 ± 1.8; *p* < 0.05) 15 m EE > 10 m EE (37.4 ± 2.3 vs. 26.8 ± 3.8; *p* < 0.01)	5 m PE + EE > 5 m EE (34.3 ± 1.8 vs. 20.6 ± 1.8; *p* < 0.01) 5 m PE + EE > 5 m PE (34.3 ± 1.8 vs. 20.7 ± 0.7; *p* < 0.01)
CD8^+^ T_N_ cells (Fig. 5b)	NS	NS	S (*F*_(3, 61)_ = 8.369; *p* < 0.0001)	5 m PE + EE > 5 m C (20.2 ± 0.8 vs. 13.4 ± 1.8; *p* < 0.05) 15 m EE > 15 m C (18.4 ± 1.8 vs. 10.2 ± 1.7; *p* < 0.05) 15 m PE + EE > 15 m C (17.6 ± 1.3 vs. 10.2 ± 1.7; *p* < 0.05)	5 m EE < 15 m EE (10.7 ± 1.1 vs. 18.4 ± 1.8; *p* < 0.05)	5 m PE + EE > 5 m EE (20.2 ± 0.8 vs. 10.7 ± 1.1; *p* < 0.01) 5 m PE + EE > 5 m PE groups (20.2 ± 0.8 vs. 10.4 ± 1.0; *p* < 0.01)
CD8^+^ T_CM_ cells (Fig. 5c)	NS	S (*F*_(2, 60)_ = 9.552; *p* < 0.001)	NS	NS	15 m C > 10 m C (12.8 ± 1.6 vs. 8.2 ± 0.6; *p* < 0.05) 15 m EE > 10 m EE (12.8 ± 2.1 vs. 8.4 ± 0.7; *p* < 0.05)	NS
CD8^+^ T_EM_ cells (Fig. 5d)	S (*F*_(6, 59)_ = 2.455; *p* < 0.05; Fig. 5d)	S (*F*_(2, 59)_ = 3.173; *p* < 0.05)	NS	NS	10 m PE > 5 m PE (1.6 ± 0.5 vs. 0.2 ± 0.1; p < 0.05) 15 m PE > 5 m PE (2.0 ± 0.6 vs. 0.2 ± 0.1; *p* < 0.05)	NS
CD8^+^ CD25^+^ cells (Fig. 5e)	NS	S (*F*_(2, 62)_ = 4.2; *p* < 0.05)	S (*F*_(3, 62)_ = 3.839; *p* < 0.05)	NS	5 m PE + EE < 10 m PE + EE (19.4 ± 3.6 vs. 33.2 ± 1.3; p < 0.05) 5 m PE + EE < 15 m PE + EE (19.4 ± 3.6 vs. 33.7 ± 1.6; *p* < 0.05)	10 m PE + EE > 10 m PE (33.2 ± 1.3 vs. 17.2 ± 6.0; *p* < 0.05)

*S* significant, *NS* not significant, *m* month, *TN* naïve T cells, *TCM* central memory T cells, *TEM* effector memory T cells

The interaction effect was non-significant for the proportion of the gated CD4^+^ T cells (Fig. 4a). However, the interaction effects were significant for CD4^+^ T_CM_ (Table 2; Fig. 4c) and T_EM_ (Table 2; Fig. 4d) cells. In addition, we observed significant main effects of (i) age for the T_N_ (Table 2; Fig. 4b), T_CM_ (Table 2; Fig. 4c), and T_EM_ (Table 2; Fig. 4d) subsets, and (ii) treatment for the T_N_ (Table 2; Fig. 4b) and T_EM_ (Table 2; Fig. 4d) subsets of CD4^+^ cells.

Post hoc analysis revealed significantly higher expression of CD4^+^ T_N_ cells in 5-month PE + EE mice compared to 5-month control mice, and 10-month and 15-month PE + EE mice (Table 2; Fig. 4b). Similarly, the proportion of CD4^+^ T_CM_ cells was significantly higher in 5-month EE and PE mice compared to 5-month control mice, 5-month PE + EE mice, and respective EE and PE mice at 10 and 15 months (Table 2; Fig. 4c). The results were, however, different for CD4^+^ T_EM_ cells (Table 2; Fig. 4d). The 5-month control and PE + EE mice expressed significantly higher CD4^+^ T_EM_ cells in the cervical lymph nodes than similar-aged EE and PE mice. Furthermore, the expression of CD4^+^ T_EM_ cells in the EE group at 5 months was significantly lower than EE group at 15 months. Likewise, the PE group at 5 months showed significantly lower expression of CD4^+^ T_EM_ cells than PE groups at both 10 and 15 months.

We observed a significant interaction effect for CD4^+^ CD25^+^ T cells (Table 2; Fig. 4e). On post hoc analysis, the 5-month PE group showed a significantly higher proportion of CD4^+^ CD25^+^ T_EM_ cells than 10- and 15-month PE groups. Additionally, we observed that 5-month-old PE mice had significantly more CD4^+^ CD25^+^ cells when compared to the same age controls.

Two-way ANOVA found significant interaction effect, as well as significant main effects of age and treatments for the proportion of CD8^+^ T cells (Table 2; Fig. 5a), with a proportion of CD8^+^ T cells significantly higher in (i) 4-month PE + EE group compared to the same age EE and PE groups, (ii) 14-month EE group compared to the 14-month controls group, and (iii) 14-month EE group compared to the 4- and 9-month EE groups. Furthermore, we observed the significant interaction effect for CD8^+^ T_EM_ cells (Table 2; Fig. 5d), significant main effects of age for CD8^+^ TC_M_ (Table 2; Fig. 5c) and T_EM_ (Table 2; Fig. 5d) cells, and significant main effect of treatment for CD8^+^ T_N_ cells (Table 2; Fig. 5b).

On post hoc analysis, significant differences were observed for CD8^+^ T_N_ cells between the treatment groups (Table 2; Fig. 5b). The 5-month PE + EE group had a significantly higher proportion of CD8^+^ T_N_ cells compared to 5-month controls, EE, and PE groups. Similarly, the 15-month EE and PE + EE groups showed a significantly higher proportion of CD8^+^ T_N_ cells than 15-month controls group. Furthermore, the 5-month EE group had a significantly less proportion of CD8^+^ T_N_ cells compared to 15-month EE group. However, the proportion of CD8^+^ T_CM_ cells was significantly higher in control and EE groups at 15 months than at 10 months (Table 2; Fig. 5c). In the case of CD8^+^ T_EM_ cells, both 10- and 15-month PE groups showed a greater proportion of cells than the 5-month PE group (Table 2; Fig. 5d).

We found a non-significant interaction effect but significant main effects of both age and treatment for CD8^+^ CD25^+^ cells (Table 2; Fig. 5e). The post hoc analyses revealed that 5-month PE + EE group had a significantly lower proportion of CD8^+^ CD25^+^ T cells than both 10- and 15-month PE + EE groups. Additionally, the 10-month PE + EE group showed a significantly higher proportion of CD8^+^ CD25^+^ T cells than the 10-month PE group.

## Discussion

In this study, we evaluated the effects of short-term cognitive stimuli and physical activity treatments alone or in combination at three different ages, i.e., 5, 10, and 15 months, on microglia and astrocyte numbers in the dentate gyrus and change in the proportion of peripheral CD4^+^ and CD8^+^ T cells, their subsets (T_N_, T_CM_, T_EM_), and CD25^+^ T cells. We observed that while all three treatments reversed the aging-related decrease in the number of immunopositive microglial cells at middle age, none of them showed any effects on the number of immunopositive astroglial cells at all ages. Additionally, all three treatments were found to modulate the proportion of peripheral T cell subsets during normal aging, especially at early and late-middle age.

### Only Short-Term EE Enhanced Microglial Number at Middle Age, Reversing the Effects of Aging. None of the Treatments Affected Astroglia Number

We observed a significant increase in the number of immunopositive IBA1^+^ microglia after short-term EE at both 5 and 10 months and after short-term PE and PE + EE at 10 months. None of the treatments significantly differed from controls at 15 months. We noticed that the significant increase observed after EE, PE, and PE + EE at 10 months could, in turn, be the result of unusual reduction in the number of IBA1 + microglia in 10-month-old control mice. Indeed, 10-month controls also showed a significantly lower number of IBA1^+^ microglial cells than 5- and 15-month controls. These results are, however, in line with previously published findings where short-term EE significantly increased microglia antigen (IBA1^+^) expression within the dentate gyrus of adult male Sprague–Dawley rats (Williamson et al. 2012). In addition, we observed that short-term PE + EE mice had significantly more IBA1^+^ microglial cells in the dentate gyrus at 15 months compared to 5- and 10-month PE + EE mice.

It must, however, be noted that the phenotype of microglia determines the functional state of microglia. Quiescent microglia within the CNS lack phenotypical markers required for antigen presentation but assist in neuronal migration and repair, recycling of neurotransmitters, regulating ion balance and buffering pH, and maintaining neuronal homeostasis (Singhal and Baune 2017). When activated in the presence of pathogens or stress proteins, microglia quickly proliferate and express major histocompatibility complex (MHC) class I and II proteins, receptors for various cytokines, toll-like receptors, Nod-like receptors, and antigens for T cell subsets essential to mounting an innate immune response (reactive microglia). The number of reactive microglia increases in areas of neurodegeneration in aging rodents (Singhal and Baune 2017), which has, in turn, been associated with aging-related cognitive and memory impairment (Rozovsky et al. 1998; Sugaya et al. 1996), depression (Norden and Godbout 2013), and neurodegenerative diseases such as AD (Mrak and Griffin 2005). The immunohistochemical stain that we used stained both quiescent and reactive microglia equally; hence, differentiating the two types of microglia was not possible. Also, we investigated mice that aged from early to late-middle age, and not old age, in a relatively stress-free environment, thereby reducing the likelihood of pathogen- and damage-associated molecular patterns (PAMPs and DAMPs) accelerating the microglia-related process. As such, an increase in microglial number during our work is indicative of the effects of normal aging with other external confounders significantly filtered out, and could primarily be due to an increase in the number of quiescent and not reactive microglia. Future research investigating the change in the proportion of two phenotypes after the three treatments is required. This is important especially when the available evidence suggests that altered microglial phenotype modulates affective-like and cognitive behaviors in young and adult rodents (Biscaro et al. 2012; Li et al. 2014; Moraes et al. 2015; Wang et al. 2018). Nonetheless, the depletion in microglial number has also been shown to protect mice for neuroinflammation-associated learning and memory impairment in postoperative young mice (Feng et al. 2017). Likewise, elimination of microglia has shown to improve cognition in six-month-old C56BL/6 mice following cranial irradiation (Acharya et al. 2016). These evidences suggest that change is microglial number may also result in change in behavior.

The overexpression of microglia can result in a significant increase in the production and expression of proinflammatory cytokines (e.g., TNF-α, IL-1β) and neurotoxic substances (e.g., reactive oxygen species, nitric oxide) (Singhal and Baune 2017), subsequently leading to cognitive dysfunction and psychiatric illnesses, such as depression (Patel 2013) and AD (Mrak 2012). However, the increase in the number of microglia may not corroborate fully with the change in microglial functions in the absence of an analysis of microglial activation state. Moreover, we observed no significant change in the microglia number at 15 months after PE and EE. Interestingly, the combination of PE and EE also showed no effects on the increase in microglia number when compared with controls at all three age points. The beneficial effects of the short-term EE on locomotor activity, anxiety-like behavior, and spatial memory at late-middle age that we have reported recently (Singhal et al. 2019) could possibly be due to an increase in the number of quiescent microglia. These results, however, do not explain the reported spatial learning impairment and depressive-like behavior at an early age after short-term PE. Yet, these results are interesting given the microglia are considered the primary immune effector cells in the brain and hence any change in microglial number could be associated with microglial dysfunctioning.

None of the short-term treatments differed significantly from controls for GFAP^+^ astroglial cells at any of the ages. However, 15-month PE mice showed significantly lower expression of GFAP^+^ astroglial cells than 5-month PE mice. It has been reported that during normal aging of the brain, astrocytes reduce in number but their phenotype changes to those of reactive astrocytes, which are the hallmarks of neuroinflammatory disorders (Clarke et al. 2018). These astrocytes affect several neural functions, such as neurotransmission, synaptic plasticity, and neurogenesis, dysregulation of which, in turn, has been associated with the development of psychiatric symptoms (Clarke et al. 2018). Indeed, the association between microglia and astrocytes during old age results in impairment of several vital neuroprotective functions, in particular, the formation and maturation of synapses, neuroinflammation, and neurodegeneration (Dilger and Johnson 2008; Moranis et al. 2012; Wang et al. 2011). As such, we hypothesized a decrease in the number of immunoreactive astrocytes within the dentate gyrus during normal aging. Our results suggest that normal aging until late-middle age has no significant effects on the number of GFAP^+^ astroglial cells in the dentate gyrus. Similarly, no effects of short-term PE, EE, and PE + EE compared to controls were observed on GFAP^+^ astroglial number in the dentate gyrus. Furthermore, although the GFAP^+^ astroglial cell number reduced in all 15-month treatment groups when compared to 5-month treatment cohorts, the reduction was significant only in case of short-term PE mice, thereby suggesting that the short-term PE might influence astrocyte-related processes during old age more than short-term EE. Perhaps, the longer duration of PE, EE, and PE + EE treatments may be required to observe a significant change in the GFAP^+^ astroglial cell number in the dentate gyrus.

Together, the results for microglia and astrocytes suggest that any short-term change in behavior or memory may not be accompanied by a concurrent change in glial cell expression and other immune and non-immune factors may be responsible for the reported functional changes (Singhal et al. 2019).

### Short-Term PE and EE Did Not Affect the Cervical Lymph Nodes CD4^+^ T Cell Subsets Until Late-Middle Age. Only Short-Term PE + EE Enhanced CD4^+^ T_N_ Cell Proportion at an Early Age

Ours is the first study to investigate the change in the proportion of T cell subsets in the cervical lymph nodes in response to short-term EE. The CSF drains to the cervical lymph nodes through the cribroid plate and nasal mucosa, and hence, the analysis of T cells from the cervical lymph nodes provides a good measure of the T cell-mediated immunity in the brain (Engelhardt et al. 2016; Goldmann et al. 2006). There were no significant differences found in the overall proportion of CD4^+^ T cells after short-term treatments at all ages suggesting no effects of the short-term physical exercise or enrichment on helper T cells. These findings are similar to a male CS mouse study whereby no effect of EE when compared with controls was found on the proportion of CD4 + T cells (Marashi et al. 2003). However, this is in contrast to a significant reduction in peripheral blood CD4 + helper T cell proportion in comparison with controls in rats which underwent short-term (4 weeks) PE using a swimming protocol (Kaufman et al. 1994). Similarly, in another rodent study, the combination of PE + EE was seen to affect T helper cells. In this case, the reduction in CD4 + T helper cell proportions as found in adolescent rat offspring in response to gestational stress was reversed with a short-term PE + EE intervention (Laviola et al. 2004). However, moderate endurance training on a treadmill has shown to increase the percentage of CD4 + T cells in the Thymus of adult rats, suggesting that the different type of exercise may elicit differnet effects on T cell subsets in different immune locations (Ferry et al. 1992). It is important to note that differences in genotypes and species across studies mentioned could also contribute to differences in reported findings of peripheral lymphocyte phenotypes.

Further subset analyses of the proportion of CD4^+^ T cells were carried out. The estimation of the T cell subsets is important in measuring the cell-mediated immune response (Golubovskaya and Wu 2016; Lanzavecchia and Sallusto 2000; Sallusto et al. 1999; Seder and Ahmed 2003). We observed a significantly higher proportion of T_N_ cells in the 5-month PE + EE mice compared to 5-month controls and PE + EE mice at 10 and 15 months. Moreover, the proportion of T_CM_ cells was significantly higher in 5-month EE and PE mice when compared to 5-month control mice, and 10- and 15-month mice that received the same treatments. Interestingly, the proportions of T_EM_ in 5-month PE and EE groups were significantly lower than the same age controls as well as older mice receiving the same treatments. Overall, this suggests that while the combination of short-term PE + EE increased the proportion of CD4^+^ T_N_ cells, PE or EE alone did not. On the other hand, short-term PE and EE in isolation not only induced an increase in the proportion of CD4^+^ T_CM_ cells but also reduced the proportion CD4^+^ T_EM_ cells at an early age.

Naïve T cells become activated, proliferate, differentiate into memory and effector cells, and show upregulation of surface marker CD25^+^ on encountering a cognate antigen. Studies investigating T cell receptor responses indicate CD4^+^ T_N_ cells proliferate and convert into memory cells at a lower rate after an initial lag period upon antigen stimulation, in comparison to CD8^+^ T_N_ cells (Foulds et al. 2002; Jelley-Gibbs et al. 2000). Similarly, the effects of aging and subsequently of PE on T cell subsets in Spleen and Thymus have been demonstrated by another study (Woods et al. 2003). The authors reported significantly higher percentage of splenic memory cells and a lower percentage of näive cells in both the CD4^+^ and CD8^+^ T cell subsets in 18-month-old BALB/c mice, which was reversed by moderate exercise training of 4 months on a treadmill. No effects of PE was observed on the T cell subsets in Thymus. In contrast, in our study, exposure to either physical or environmental enrichment appears to induce an earlier expansion of CD4 + T_N_ to CD4^+^ T_CM_ cells in the cervical lymph nodes; however, it must be noted that mice during our study aged only until middle age. Also, the lower proportions of CD4 + T_EM_ cells in 5-month PE and EE can be explained by the fact that mice during our experiments were raised in a relatively stress-free environment also devoid of PAMPs. However, aging can also result in the production of DAMPs, which can act as cognate antigens. This might explain the higher proportions of CD4 + T_EM_ cells at 10 and 15 months.

The controlled environment could also be the reason for no differences noted in the proportion of CD4^+^ CD25^+^ cells except for 5-month PE group showing a significantly higher proportion of CD4^+^ CD25^+^ cells than the 5-month controls and the older mice receiving the same treatment. CD25^+^ is the alpha chain of the IL-2 receptor and is upregulated following T cell receptor ligation acquiring high affinity and making cells sensitive for IL-2, also acting as early T cell activation marker. IL-2 controls the differentiation and homeostasis of T cells, thereby regulating cell-mediated immune response. CD4^+^ CD25^+^ cells may be associated with the ‘activation,’ ‘priming,’ or ‘memory’ state of CD4^+^ cells (Sakaguchi 2000). Also, since CD4^+^ CD25^+^ cells have been reported to suppress proliferation of effector T cells (Sakaguchi et al. 2008; Wing and Sakaguchi 2008), this suggests that short-term PE might help to develop immunological self-tolerance at an early age.

### Short-Term EE and PE + EE Increase the Proportion of Peripheral CD8^+^ T_N_ Cells in Young and Late-Middle Age Mice, Respectively. Only PE + EE Increases the Proportion of Early-Activated CD8^+^ T Cells Across All Ages

We observed that the proportion of peripheral CD8^+^ T cells at the late-middle age increased significantly after short-term EE. However, no change in the proportion of CD8^+^ T cells was noted after both short-term PE and PE + EE at all ages. This was contrary to the hypothesis that short-term PE will improve neuroimmune functions during normal aging by reducing the proportion of CD8^+^ T cells in C57BL/6 mice in a controlled environment, as has been reported in the spleens of rodents trained in PE using a swimming protocol over 4 weeks (Kaufman et al. 1994). Similarly, the reduced anxiety-like behavior and improvement in spatial memory at middle and late-middle age, respectively, after short-term EE (Singhal et al. 2019) is not explained by CD8^+^ T cell results.

CD8 + T_N_ cells require significantly less time of antigen exposure for activation than CD4 + T_N_ cells. This, in turn, translates into the less time required by the CD8 + T_N_ cells to activate, divide and differentiate, and have a faster rate of division than CD4 + T_N_ cells (Seder and Ahmed 2003). Furthermore, CD4 + effector T cells are more susceptible to apoptosis at the end of the effector phase than CD8 + effector T cells. This provides a better opportunity to CD8 + effector T cells to develop into stable long-term resting memory cells than CD4 + effector cells. Also, unlike CD4 + memory cells, both CD8 + memory cell subsets (T_CM_ and T_EM_) are equally efficient in producing effector functions on restimulation with antigen. These differences makes CD8 + T cells more potent than CD4 + T cells in dispersing cell-mediated immune functions.

Further subset analyses of the proportion of CD8^+^ T cells revealed an increase in proportion of CD8^+^ T_N_ but not T_CM_ and T_EM_ cells after short-term EE at late-middle age. However, a human study has shown that aging is associated with a decrease in CD8^+^ T_N_ cells in donor cervical lymph nodes (Lazuardi et al. 2005). This suggests that EE improves basal cytotoxic immunity during the late-middle-age reversing the age-related effects. This, possibly also explains the reported improvement in brain functions after short-term EE (Singhal et al. 2019). In contrast, we observed that the combination of short-term PE and EE also increased the proportion of CD8^+^ T_N_ cells, but only at an early age and not at middle and late-middle age. These results are interesting and show the influence that the inclusion of a running wheel in EE paradigms can have on cell-mediated immunity. Furthermore, the proportion of CD8^+^ T_CM_ cells in the cervical lymph nodes of control mice significantly increased from middle to late-middle age. This can again be explained by the fact that there is an increase in the production of DAMPs during aging, which may act as cognate antigens stimulating the conversion of the peripheral CD8^+^ T_N_ cells to CD8^+^ T_CM_ cells. The latter have little or no effector function but mediate reactive memory where they migrate to secondary lymphoid organs, and some of them proliferate and differentiate into effector cells in response to antigenic stimulation, thus helping the body to resolve inflammation (Liao et al. 2011). The proportion of CD8^+^ T_EM_ cells in the cervical lymph nodes of 5-month PE mice was significantly lower compared to older PE groups. Since the CD8^+^ T_EM_ cells are important mediators of protective memory as mentioned before, the lower number of CD8^+^ T_EM_ cells after PE at 5 months suggests that perhaps the cell-mediated immunity gets compromised after short-term PE at an early age, which may have a role to play in the reported development of depressive-like behavior in young mice (Singhal et al. 2019).

The combination of short-term PE with EE significantly improved the proportion of early-activated CD8^+^ CD25^+^ T cell from middle age onwards when compared to early age PE + EE mice, suggesting that PE + EE might enhance CD8 + T cell responsiveness if provided in combination and for long-term (Backer et al. 2018). Further research into investigating the effects of long-term PE, EE, and their combination on aging-associated changes in T cell subset proportions is required.

## Limitations of the Study and Future Directions

We conducted behavioral testing during the last three weeks before collecting brain samples for IHC and FACS. As such, there is a possibility that other factors, such as social or handling stress (Hardy et al. 1990; Stefanski 2001; Stefanski and Engler 1999) and sensory stimulation (Roberts 1995, 2000), could have modulated glia and T cell subset numbers. The composition of EE objects, i.e., wood or plastic, soft or hard, may also have a significant impact on the observed results. These variables must be explored in detail in future research investigating the effects of PE and EE on neuroimmune mechanisms. Furthermore, it is the primed phenotype of glial cells that drives neuroinflammation and the subsequent functional changes. This encompasses much more than an increase in the number of immunopositive glial cells in the dentate gyrus. Hence, examining the morphology of glial cells (quiescent and reactive microglia) and levels of other activation markers, for example cytokines, chemokines, C-reactive protein, and protein kinases, is required to develop full understanding of an inflammatory phenotype after the three treatments. T cells from cervical lymph nodes are good representatives of T cells in the brain. However, comparison between the T cells obtained from other key regions, such as blood, thymus, and cerebrospinal fluid, is required to develop better understanding. Our immune results, therefore, do not make a full profile of the inflammatory phenotype during normal aging and may not fully explain the reported change in brain functions after the three treatments (Singhal et al. 2019). However, we have provided a foundation towards developing understanding on the role of PE and EE in the modulation of brain glial cells and peripheral T cells.

## Conclusion

Our study is the first to investigate the change in the number of brain glia cells and T cell subsets within the cervical lymph nodes of immunologically unchallenged mice in response to short-term PE, EE, and PE + EE across the lifespan. Our findings suggest that both short-term PE and EE elicit neuroimmune changes during normal aging. We noted that the effect of short-term EE alone on glial cells is stronger. Furthermore, both short-term PE and EE enhance cytotoxic immune potential by increasing the proportion of CD8^+^ T_N_ cells. Similarly, the combination of short-term PE and EE led to an increase in the proportion of early-activated CD8 + T cells, suggesting enhanced cytotoxic immunity. We also conclude that the neuroimmune effect of short-term PE and EE is not additive.

## Electronic supplementary material

Electronic supplementary material 1 (XLSX 18 kb)

Supplementary file2 (JPG 371 kb) Supplementary Fig. I. Representative images of the (a) PE, (b) EE and (c) PE+EE protocols.

Supplementary file3 (JPG 134 kb) Supplementary Fig. II. Representative of the density plots showing (a) Gated CD45+ cells, (b) CD3+ cells derived from gated CD45+ cells, and (c) CD3+ CD4+ and CD3+ CD8+ T cells distinguished from total CD3+ gated cells. Further gating on CD44+ and CD62L+ cell populations enabled the identification and estimation of (d) CD4+ and (e) CD8+ T cell subsets, i.e., Naïve (TN), Central memory (TCM) and Effector memory (TEM) T cells.

Supplementary file4 (JPG 122 kb) Supplementary Fig. III. Representative of density plots showing the proportion of early activation markers CD25+ on (a) CD4+ and (b) CD8+ T cell subpopulations derived from the gated CD45+ T cells.

Electronic supplementary material 5 (DOCX 22 kb)
