# Supplementary figures and images for: Short-Term Environmental Enrichment is a Stronger Modulator of Brain Glial Cells and Cervical Lymph Node T Cell Subtypes than Exercise or Combined Exercise and Enrichment
Source: Cell Mol Neurobiol. 2020 May 25;41(3):469–86. doi: 10.1007/s10571-020-00862-x (PMC7920895; doi:10.1007/s10571-020-00862-x)

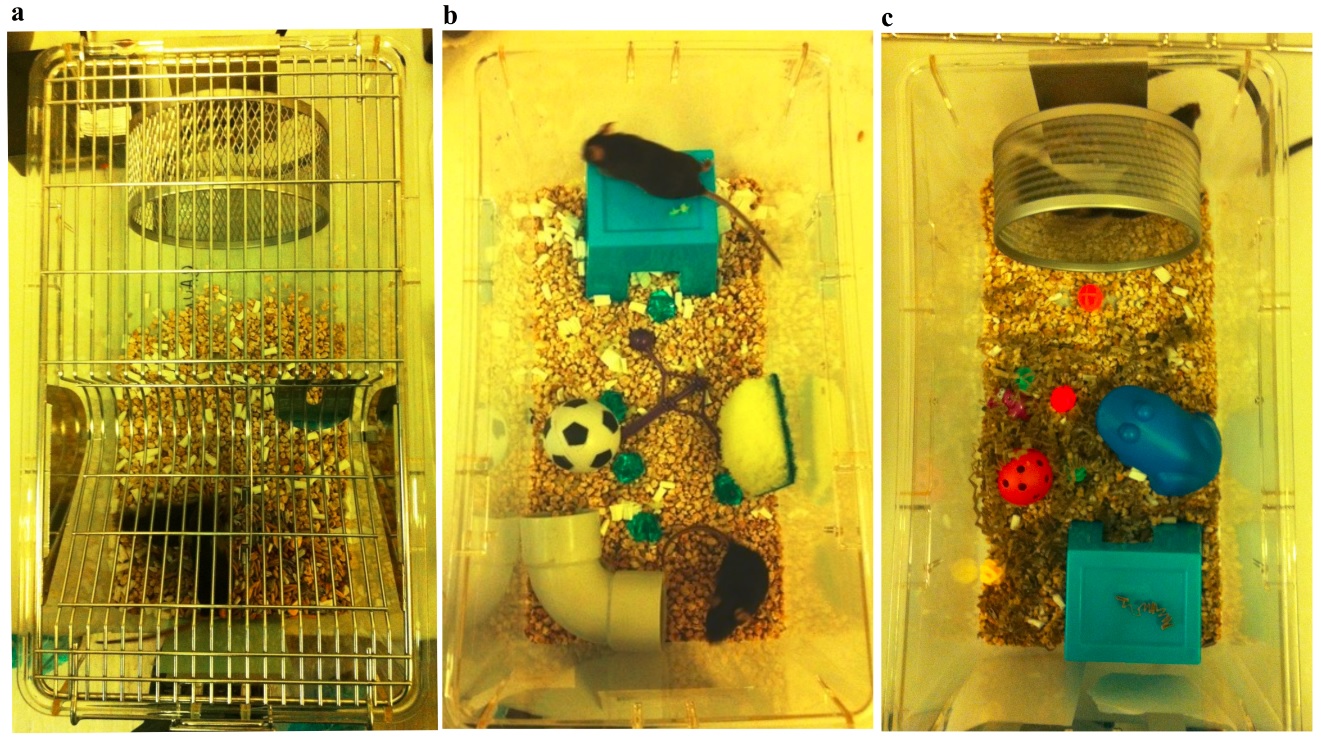

Supplement: Supplementary file 2 — Supplementary file2 (JPG 371 kb) Supplementary Fig. I. Representative images of the (a) PE, (b) EE and (c) PE+EE protocols. [file 10571_2020_862_MOESM2_ESM.jpg]

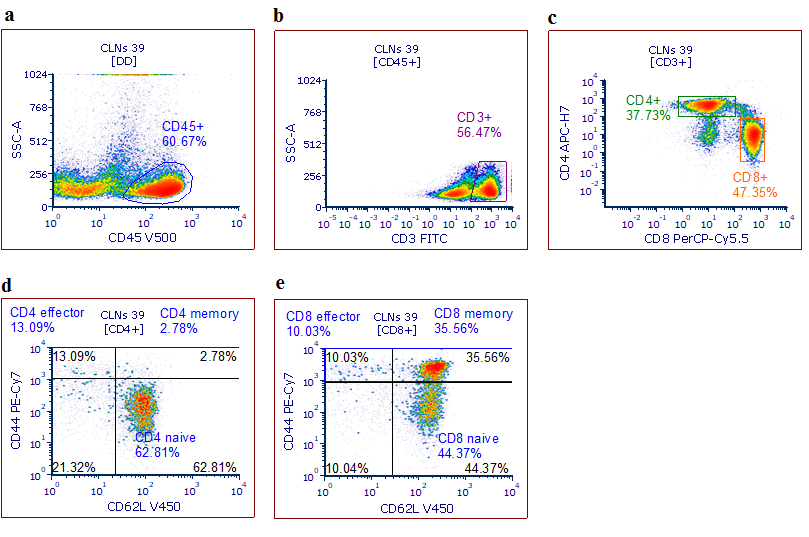

Supplement: Supplementary file 3 — Supplementary file3 (JPG 134 kb) Supplementary Fig. II. Representative of the density plots showing (a) Gated CD45+ cells, (b) CD3+ cells derived from gated CD45+ cells, and (c) CD3+ CD4+ and CD3+ CD8+ T cells distinguished from total CD3+ gated cells. Further gating on CD44+ and CD62L+ cell populations enabled the identification and estimation of (d) CD4+ and (e) CD8+ T cell subsets, i.e., Naïve (TN), Central memory (TCM) and Effector memory (TEM) T cells. [file 10571_2020_862_MOESM3_ESM.jpg]

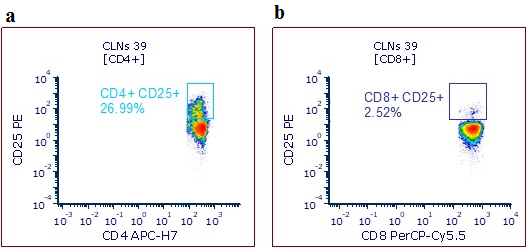

Supplement: Supplementary file 4 — Supplementary file4 (JPG 122 kb) Supplementary Fig. III. Representative of density plots showing the proportion of early activation markers CD25+ on (a) CD4+ and (b) CD8+ T cell subpopulations derived from the gated CD45+ T cells. [file 10571_2020_862_MOESM4_ESM.jpg]
