# Supplementary material for: Short-Term Environmental Enrichment is a Stronger Modulator of Brain Glial Cells and Cervical Lymph Node T Cell Subtypes than Exercise or Combined Exercise and Enrichment
Source: Cell Mol Neurobiol. 2020 May 25;41(3):469–86. doi: 10.1007/s10571-020-00862-x (PMC7920895; doi:10.1007/s10571-020-00862-x)
Supplement: Supplementary file 5 — Electronic supplementary material 5 (DOCX 22 kb) [file 10571_2020_862_MOESM5_ESM.docx]

**Supplementary Table I.** Statistical data and group comparisons for T cell subsets percentage in the cervical lymph nodes of C57BL/6 mice after short-term PE, EE, and PE+EE treatments

| **T cell phenotype** | **Interaction effect** | **Main effect of age** | **Main effect of treatment** | **Vs. Control (%)** | **Between age groups (%)** | **Between treatment groups (%)** |
| --- | --- | --- | --- | --- | --- | --- |
| CD4^+^ cells  (Fig 8A) | **NS** | **NS** | **NS** | **NS** | **NS** | **NS** |
| CD4^+^ T_N_ cells  (Fig 8B) | **NS** | **S** (F _(2, 60)_ = 7.445; p < 0.01) | **S** (F _(3, 60)_ = 3.024; p < 0.05) | 5m PE+EE > 5m C (14.6 ± 1.4 vs. 9.7 ± 1.2; p < 0.01) | 5m PE+EE > 10m PE+EE (14.6 ± 1.4 vs. 10.1 ± 0.7; p < 0.01)  5m PE+EE >15m PE+EE (14.6 ± 1.4 vs. 9.2 ± 0.7; p < 0.01) | **NS** |
| CD4^+^ T_CM_ cells  (Fig 8C) | **S** (F _(6, 62)_ = 3.497; p < 0.01) | **S** (F _(2, 62)_ = 9.335; p < 0.001) | **NS** | 5m EE > 5m C (11.1 ± 1.0 vs. 5.7 ± 1.4; p < 0.01)  5m PE > 5m C (10.3 ± 0.7 vs. 5.7 ± 1.4; p < 0.05) | 5m EE > 10m EE (11.1 ± 1.0 vs. 6.2 ± 0.4; p < 0.01)  5m EE > 15m EE (11.1 ± 1.0 vs. 5.6 ± 0.5; p < 0.01)  5mPE > 10m PE (10.3 ± 0.7 vs. 4.7 ± 1.2; p < 0.01)  5m PE > 15m PE (10.3 ± 0.7 vs. 4.6 ± 1.4; p < 0.01) | 5m EE > 5m PE+EE (11.1 ± 1.0 vs. 6.4 ± 0.5; p < 0.05)  5m PE > 5m PE+EE (10.3 ± 0.7 vs. 6.4 ± 0.5; p < 0.05) |
| CD4^+^ T_EM_ cells  Fig (8D) | **S** (F _(6, 58)_ = 3.399; p < 0.01) | **S** (F _(2, 58)_ = 4.931; p < 0.05) | **S** (F _(3, 58)_ = 3.324; p < 0.05) | 5m EE < 5m C (0.7 ± 0.3 vs. 3.5 ± 0.7; p < 0.01)  5m PE < 5m C (0.8 ± 0.4 vs. 3.5 ± 0.7; p < 0.01) | 5m EE < 15m EE (0.7 ± 0.3 vs. 2.7 ± 0.2; p < 0.05)  5m PE < 10m PE (0.8 ± 0.4 vs. 3.6 ± 0.6; p < 0.01)  5m PE < 15m PE (0.8 ± 0.4 vs. 3.4 ± 0.3; p < 0.01 | 5m EE < 5m PE+EE (0.7 ± 0.3 vs. 3.1 ± 0.4; p < 0.01)  5m PE < 5m PE+EE (0.8 ± 0.4 vs. 3.1 ± 0.4; p < 0.01) |
| CD4^+^ CD25^+^ cells (Fig 8E) | **S** (F _(6, 61)_ = 3.235; p < 0.01) | **NS** | **NS** | 5m PE > 5m C (22.7 ± 2.4 vs. 10.7 ± 2.7; p < 0.01) | 5m PE > 10m PE (22.7 ± 2.4 vs. 13.0 ± 3.4; p < 0.05)  5m PE > 15m PE (22.7 ± 2.4 vs. 12.1 ± 3.4; p < 0.05) | **NS** |
|  | | | | |  |  |
| CD8^+^ cells  (Fig 9A) | **S** (F _(6, 61)_ = 2.286; p < 0.05) | **S** (F _(2, 61)_ = 5.321; p < 0.01) | **S** (F _(3, 61)_ = 6.713; p < 0.001) | 15m EE > 15m C (37.4 ± 2.3 vs. 25.7 ± 2.9; p < 0.05) | 15m EE > 5m EE (37.4 ± 2.3 vs. 20.6 ± 1.8; p < 0.05)  15m EE > 10m EE (37.4 ± 2.3 vs. 26.8 ± 3.8; p < 0.01) | 5m PE+EE > 5m EE (34.3 ± 1.8 vs. 20.6 ± 1.8; p < 0.01)  5m PE+EE > 5m PE (34.3 ± 1.8 vs. 20.7 ± 0.7; p < 0.01) |
| CD8^+^ T_N_ cells  (Fig 9B) | **NS** | **NS** | **S** (F _(3, 61)_ = 8.369; p < 0.0001). | 5m PE+EE > 5m C (20.2 ± 0.8 vs. 13.4 ± 1.8; p < 0.05)  15m EE > 15m C (18.4 ± 1.8 vs. 10.2 ± 1.7; p < 0.05)  15m PE+EE > 15m C (17.6 ± 1.3 vs. 10.2 ± 1.7; p < 0.05) | 5m EE < 15m EE (10.7 ± 1.1 vs. 18.4 ± 1.8; p < 0.05) | 5m PE+EE > 5m EE (20.2 ± 0.8 vs. 10.7 ± 1.1; p < 0.01)  5m PE+EE > 5m PE groups (20.2 ± 0.8 vs. 10.4 ± 1.0; p < 0.01) |
| CD8^+^ T_CM_ cells  (Fig 9C) | **NS** | **S** (F _(2, 60)_ = 9.552; p < 0.001) | **NS** | **NS** | 15m C > 10m C (12.8 ± 1.6 vs. 8.2 ± 0.6; p < 0.05)  15m EE > 10m EE (12.8 ± 2.1 vs. 8.4 ± 0.7; p < 0.05) | **NS** |
| CD8^+^ T_EM_ cells  (Fig 9D) | **S** (F _(6, 59)_ = 2.455; p < 0.05; Fig 6D) | **S** (F _(2, 59)_ = 3.173; p < 0.05) | **NS** | **NS** | 10m PE > 5m PE (1.6 ± 0.5 vs. 0.2 ± 0.1; p<0.05)  15m PE > 5m PE (2.0 ± 0.6 vs. 0.2 ± 0.1; p < 0.05) | **NS** |
| CD8^+^ CD25^+^ cells  (Fig 9E) | **NS** | **S** (F _(2, 62)_ = 4.2; p < 0.05) | **S** (F _(3, 62)_ = 3.839; p < 0.05) | **NS** | 5m PE+EE < 10m PE+EE (19.4 ± 3.6 vs. 33.2 ± 1.3; p < 0.05)  5m PE+EE < 15m PE+EE (19.4 ± 3.6 vs. 33.7 ± 1.6; p < 0.05) | 10m PE+EE > 10m PE (33.2 ± 1.3 vs. 17.2 ± 6.0; p < 0.05) |

**Legend:** S: significant, NS: not significant, m: month, TN: naïve T cells, TCM: central memory T cells, TEM: effector memory T cells.
